# Efficacy of Entomopathogenic Fungal Formulations against *Elasmolomus pallens* (Dallas) (Hemiptera: Rhyparochromidae) and Their Extracellular Enzymatic Activities

**DOI:** 10.3390/toxins14090584

**Published:** 2022-08-25

**Authors:** Fredrick Fidelis Umaru, Khanom Simarani

**Affiliations:** 1 Division of Microbiology, Faculty of Science, Institute of Biological Sciences, University of Malaya, Kuala Lumpur 50603, Malaysia; 2 Department of Biological Sciences, Faculty of Science, Taraba State University, Jalingo 660213, Nigeria

**Keywords:** biological control, bioassay, enzyme, entomopathogenic fungi, oil formulation, seed bug

## Abstract

*Elasmolomus pallens* are post-harvest insect pests of peanuts that are becoming resistant to chemical insecticides. In this, we study evaluated the effect of conidial formulations on entomopathogenic fungi against *E. pallens* to reduce the adverse effects. Fungal conidia were formulated and applied on sterile filter papers at varying concentrations (1 × 10^4^–1 × 10^8^ conidia mL^−1^) inside plastic containers. The test insects were exposed and maintained in a relative humidity of 80 ± 10% for 10 d at room temperature (25 ± 2 °C). Mortality was recorded every 24 h. Dose–response bioassay (LC50 and LC90) values for *Aspergillus flavus* formulated in oil were 1.95 × 10^6^ and 3.66 × 10^9^ conidia/mL, whereas formulations in Tween 80 had 9.36 × 10^7^ and 6.50 × 10^9^ conidia/mL. However, oil-formulated *Metarhizium anisopliae* had 3.92 × 10^6^ and 2.57 × 10^8^ conidia/mL, with 6.85 × 10^6^ and 5.37 × 10^8^, for formulations in Tween 80. *A. flavus* had LT50 values of 3.3 and 6.6 days, whereas *M. anisopliae* had LT50 values of 3.6 and 5.7 d. Maximum protease, chitinase, and lipase activities of 2.51, 0.98, and 3.22 U/mL, respectively, were recorded for *A. flavus*, whereas values of 2.43, 0.93, and 3.46 were recorded for *M. anisopliae*. The investigated pathogens demonstrate potential against *E. pallens*; therefore, their applicability under field conditions requires further investigation.

## 1. Introduction

Insects from the order Hemiptera, commonly described as ‘true bugs,’ are well-recognized as economically important pests in commercial crops [1]. The test insects, *Elasmolomus pallens* (Hemiptera: Rhyparochromidae), are post-harvest insect pests [2], the family members of which are diverse and comprise the most prominent Lygaeoidea [3]. *Elasmolomus pallens* are distributed across various global climatic zones, including the tropical, subtropical, and temperate climatic zones. The insects’ distribution is linked to their appetite for the legume Arachis hypogea L. [4]. Adults and nymphs are a cause of significant economic loss of peanuts in Sub-Saharan Africa, attacking entire pods using their piercing rostrum. Peanut pods are usually attacked by insects in the field during harvest and storage. When such infestations occur, the seeds become shriveled, resulting in increased fatty acid content and a rancid flavor of the oil [5].

The menace of *E. pallens* as a pest of economic concern is often tackled using classical biological and chemical control methods. With classical biological methods, predators such as Cephalonomia (a bethylid wasp) and the Reduviidae *Coranus pallidus* are employed as valuable predators of the eggs, nymphs, and adult insects [4]. With the chemical control method, 0.5% lindane and 2% malathion are applied on peanut stalks to prevent infestation [4]. However, the tendencies for this pest species to attack crop plants are evident, and the negative impacts of synthetic chemicals on biodiversity, the environment, and health are significant. Therefore, lindane is no longer in use. It has been asserted that commonly used synthetic insecticides, such as organophosphates, promote harmful environmental hazards [6].

A report by Sharma et al. [7] reveals that the chemical method of control has produced resistance among insect populations, toxic effects on the environment, and traces of insecticide residues in food and food products. The continuous use of chemical insecticides on hemipteran bugs may lead to resistance, considerably impacting food security and safety, with negative effects on natural enemies [8]. It is essential to search for alternative environmentally friendly biological control measures, considering the lethal effects of chemical pesticides on the environment [9].

Entomopathogenic fungi are naturally pathogenic to insect hosts, harmless to humans, good regulators of the insect population in the natural environment, and impose no adverse effects on the environment [10]. Entomopathogenic fungi vary in their specificity to natural hosts that they infect and are often referred to as specialist or generalist pathogens [11].

Unlike pathogens such as protozoa, bacteria, and viruses, which a host must ingest to initiate infection, entomopathogenic fungi have well-developed mechanisms that exploit nutrients on the insect cuticle, aiding in host colonization and infection. This makes entomopathogenic fungi a choice pathogen for the biological control of sucking *E. pallens* [12]. There are existing reports on the use of entomopathogenic fungi for the biological control of insects from the order Hemiptera. However, there is currently a lack of reports on their application as biological control agents of *E. pallens*. Although *A. flavus* is perceived as a harmful pathogen to humans and animals, given its ability to infest and produce aflatoxins on agricultural grain products [13], several reports have emphasized and described it as a ubiquitous mold, and not all of its strains can produce aflatoxins [14,15]. Additionally, atoxigenic *A. flavus* strains are known to be used for the biological control of toxigenic strains in crop plantations via competitive bio-exclusion to prevent aflatoxin contamination in grains [16].

Furthermore, proper formulations containing the required carriers are essential for host infection during large-scale applications of fungal entomopathogens [17]. Studies demonstrated that oil-based formulations enhanced the adhesion of conidia, conferred protection from ultraviolet radiation, and increased the virulence of entomopathogenic fungi on insect hosts compared to aqueous formulations [17].

In nature, some isolates of entomopathogenic fungi (EPF) possess the capacity to kill their host faster than others. The ability to secrete hydrolytic cuticle-degrading enzymes confers the property of virulence or nonvirulence. To eliminate their hosts, they must breach the epicuticle via specific and non-specific events [18]. The synthesis of a cocktail of extracellular hydrolytic cuticle-degrading enzymes, including proteases, chitinases, lipases [19], catalase, and phospholipase C [20] by EPF forms the underlying basis for the infection of insects, aiding in the degradation of host cuticle and penetration of the hemocoel [21,22]. Some of these hydrolytic cuticle-degrading enzymes (CDEs) are determinants of virulence for EPF [23] and work in concert to breach the proteo-chitin matrix to enable penetration into the hemolymph and when exiting to the surface of the cadaver for conidiation [24].

Previous studies by Dong et al. [25] and García-Munguía et al. [26] reported that entomopathogenic fungi conidia applied on filter paper were highly effective at infecting and killing the mosquito *Aedes aegypti* via horizontal propagule transmission during sexual activities. However, the question of the virulence of formulated propagules applied on filter paper surface to which *E. pallens* is exposed remains to be answered. Therefore, it is essential to establish and validate the novelty of horizontal transmission of entomopathogenic fungal propagules against insects such as *E. pallens*, which belong to a completely different order. Hence, the aim of this study was to determine the virulence of the formulated entomopathogenic fungi against *E. pallens* when applied on sterile filter paper surfaces.

## 2. Results

### 2.1. Fungal Isolates

The colony morphology of *A. flavus* showed dense sporulation on the culture medium. The colony color appeared dirty green, with septate hyphae, naturally radiate phialides (7.5–12 × 3.5–4.2 µm), and classically globose-to-*subglobose vesicles* (19–45 µm). The conidial size ranged between 3.5–5.2 × 2.2–2.8 µm. *M. anisopliae* produced dark-herbage green colony colors, with cylindrical and podgy phialides. Its spores were colorless and ellipsoidal with a rounded apex and slightly truncate base, whereas the conidia size ranged between 4.8–6.1 × 2.2–3.6 µm, respectively.

### 2.2. Dose–Response Bioassay

**Figure 1:**
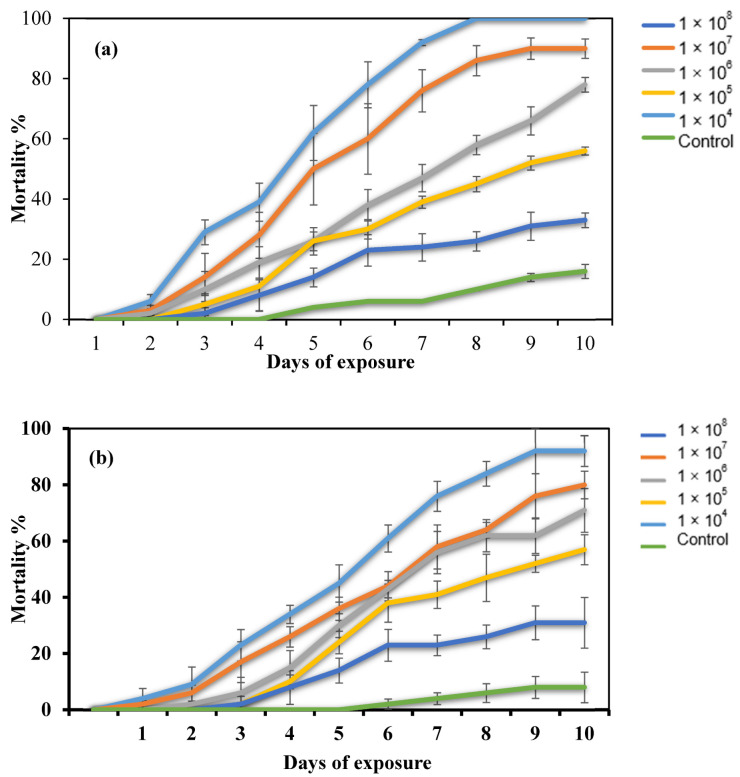
Mortality of *E. pallens* exposed to different concentrations of *A. flavus* isolate. (**a**) Formulation in oil and (**b**) formulation in 0.05% Tween 80. Mortality data were corrected using Abbott’s formula.

Mortality increased in the dose–response bioassay with increased conidial concentration (Table 1). The two fungal isolates generated significantly different mortality rates against *E. pallens* (F5, 60 = 5.745; *p* < 0.05). Additionally, oil-formulated conidia showed enhanced virulence compared to the formulation in the surfactant, Tween 80 (Table 1). The highest conidial concentration (1 × 10^8^ conidia/mL) of *A. flavus* isolates formulated in oil achieved 100% cumulative mortality after 8 days. In contrast, the same conidial concentration formulated in Tween 80 achieved 92% cumulative mortality after 9 d of treatment (Figure 1). The mortality rate was substantially higher in the conidial concentrations used compared with the control (F = 4.394; df = 5, 60; *p* < 0.05). The insect mortalities in the control were less than 10% after 10 d.

**Figure 2:**
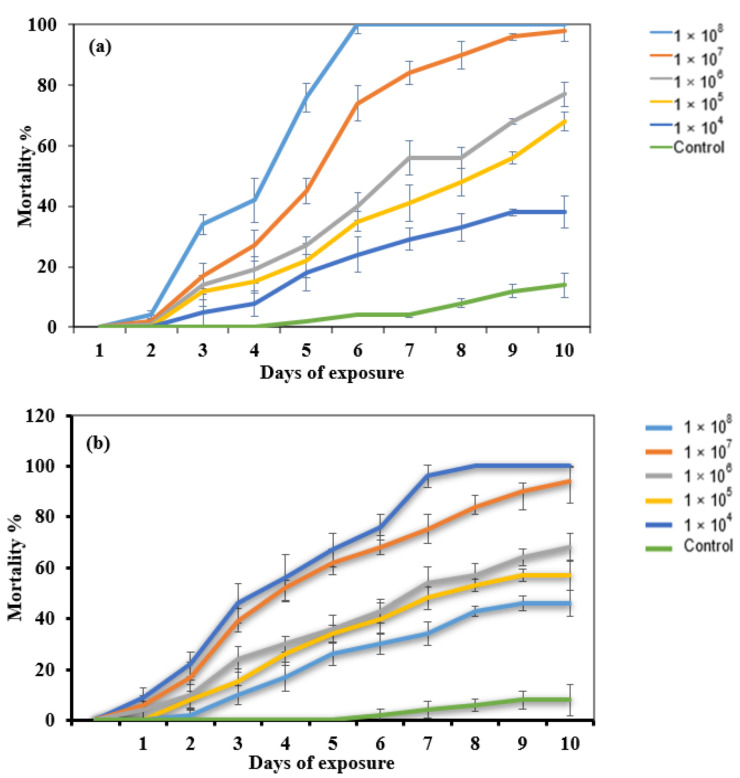
Mortality of *E. pallens* exposed to different conidia of *M. anisopliae*. (**a**) Formulation in oil and (**b**) formulation in 0.05% Tween 80. Mortality data were corrected using Abbott’s formula.

The dose–response bioassay involving *M. anisopliae* showed effects on the bugs’ mortalities based on the formulation type. *M. anisopliae* conidial concentration (1 × 10^8^ conidia/mL) formulated in oil achieved a 100% cumulative mortality in 6 d after treatment, whereas a 100% cumulative mortality for the same conidial concentration formulated in 0.05% Tween 80 was achieved after 8 d of treatment. This shows variations in the efficacies of the fungal conidial formulations. The mortality of the bugs was faster for the oil-formulated conidia compared to the formulations in Tween 80, demonstrating the enhanced efficacy of oil-formulated conidia. Significant mortalities of *E. pallens* due to *M. anisopliae* were observed (F5, 60 = 4.363; *p* < 0.05), whereas the mortalities in the control never exceeded 10% (Figure 2). Therefore, it is possible that control mortality could become zero or be set at zero when Abbott’s correction results in negative mortality. In the case of this study, no negative mortality was obtained.

**Figure 3:**
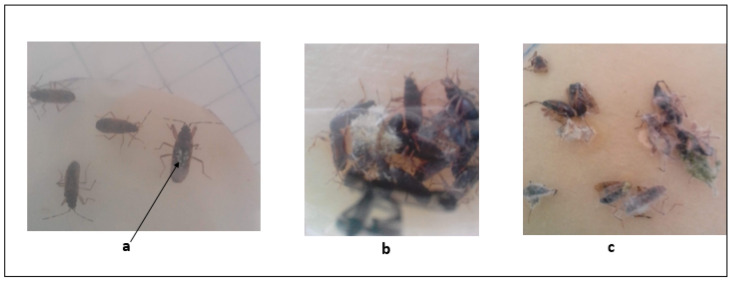
Mycotic infection of *E. pallens* results in dead and fungal development on the dead cadavers. (**a**) Infected bug showing fungal mycelia growing on its surface, (**b**) *A. flavus* mycelia growing on the cadavers of *E. pallens*, and (**c**) *M. anisopliae* mycelia growing on cadavers of *E. pallens*.

The infected bugs showed visible mycosis characterized by the development of fungal mycelia on the integument while still alive. In this stage, the insects became sluggish, losing the ability to copulate and leading to the onset of death. Dead bug cadavers cultured on damp sterile filter paper developed fungal mycelial characteristics of the isolates used in the study (Figure 3).

Under the bioassay conditions, the mean mortalities of the bugs exposed to individual conidial concentrations of the isolates were compared (Table 1). Mean mortality values after 10 d of inoculation were proportional to the fungal conidial concentrations (108 and 107) used for each isolate. The mean mortality values for oil-formulated conidia were observed to be higher (F = 4.882; df = 5,60; *p* < 0.05) compared to conidia formulated in Tween 80. This shows that the virulence of the fungal pathogens was enhanced when formulated in oil relative to that of pathogens formulated in Tween 80.

Mortalities 10 d after inoculation differed significantly depending on the dose (108–104 and control) for both isolates formulated in oil (*A. flavus* BAMF2a F = 5.60; df = 5, 60; *p* < 0.05; *M. anisopliae* F = 8.825; df = 5, 60; *p* < 0.05) and 0.05% Tween 80 (*A. flavus* isolate BAMF2a F = 4.3934; df = 5, 60; *p* < 0.05; *M. anisopliae* F = 4.363; df = 5, 60; *p* < 0.05) (Table 1). The results show that the mortalities observed in the bioassay were dose-dependent.

The bioassay experiment determined the lethal concentrations (LC50 and LC90) and lethal time (LT50 and LT90) for the fungal isolates. We observed that the LC and LT values were lower for fungal conidia formulated in oil than in Tween 80 (Table 2 and Table 3). However, comparisons between the two formulations indicate that conidia formulated in oil showed increased virulence relative to that formulated in Tween 80. The *A. flavus* isolate formulated in oil sometimes performed better than *M. anisopliae*. The comparison was made by calculating the 95% confidence interval (CI) for each population. If the CIs do not overlap, a significant difference exists. In contrast, if CIs overlap, we concluded that no significant difference existed. The CIs do not overlap; hence, they are different.

*M. anisopliae* formulated in oil killed the bugs at an LC50 and LC90 of 3.92 × 10^6^ and 5.37 × 10^8^ conidia/mL (Table 2), respectively, producing LT50 and LT90 values of 3.6 and 5.7 d, respectively (Table 3). These values were found to be lower compared to results obtained for fungal conidia formulated in Tween 80.

### 2.3. Enzyme Assay

The enzyme activity of the isolates was observed to increase with days of culture, with activity later decreasing (Table 4). Maximum protease activity for *A. flavus* isolates (2.51 U/mL) was obtained on the 8th day, whereas *M. anisopliae* (2.43 U/mL) was observed on the 6th day. For the chitinase enzyme, maximum activity (0.98 U/mL) was recorded for the *A. flavus* isolate on the 8th day, and *M. anisopliae* also exhibited maximum chitinase activity (0.93 U/mL) on the 8th. Nevertheless, maximum lipase activity (3.22 U/mL) was obtained for *A. flavus* isolate on the 6th day compared to that obtained for *M. anisopliae* (3.46 U/mL).

### 2.4. Correlation between Rates of Mortality and Enzyme Activities

The mortality rate data of *E. pallens* were analyzed in correlation with the enzymatic activity of the fungal pathogens (Table 5). The correlation coefficient (*r*) achieved for the mortality of *E. pallens* and cuticle-degrading enzymes (protease, chitinase, and lipase) exhibited positive correlations for the two isolates.

## 3. Discussion

Entomopathogenic fungi are valuable alternatives to conventional synthetic insecticides and are broadly applied to effectively manage agroecosystem pests [27]. Compared to other microbial pathogens, they confer several advantages when applied for pest control [28]. Although insects have established myriads of defenses against infective pathogens, pathogens have likewise coevolved to counter these defenses [27].

The present study confirms that the two fungal isolates of *A. flavus* isolates and *M. anisopliae* are potential biocontrol agents for the management of the seed bug *E. pallens*. The mortalities of the bugs were found to be proportional to the conidial concentrations used in the bioassay involving the two fungal isolates. Conidia of both isolates formulated in oil showed increased virulence relative to those formulated in Tween 80 surfactant, as evidenced by the mortality rates and the number of positive cadavers confirmed upon culture. Oil-based formulations of biopesticides have, over the years, been found to enhance the adherence of pathogenic fungal propagules to insect cuticle, promote the spread of the propagules over the insect body, penetration of the insect integument, protection from the effect of ultraviolet (UV) light radiation, and enhanced infectivity of the propagules, even under low moisture (humidity) conditions [9,29,30]. Furthermore, vegetable-oil-based formulations, compared to water-based formulations, have been reported to demonstrate increased virulence against various hosts, including *Bemisia tabaci* [31], arid locust, Schistocerca gregoria under low humidity [32], and ticks [29,33,34].

The efficacy of *A. flavus* was previously studied against mango seed weevil, *Sternochetus mangiferae* (Coleoptera: Curculionidae), producing mortality rates of 80% at a conidial concentration of 6.8 × 10^7^ conidia/mL. Aspergillus species have been reported to kill the tropical locust *Zonocerus variegatus* [35] and cause infection among many insect populations. However, it is unknown whether it is host-specific. Previous studies have reported the effectiveness of *A. flavus* against a wide range of insects [27,36,37]. *A. flavus* was also tested against *Galleria mellonella*, killing 100% of insects after 48 h when injected with 3 × 10^3^ conidia/mL of the fungus. In contrast, *Aspergillus fumigatus* and *Aspergillus nidulans* lacked parasitic attributes [38].

We observed that *M. anisopliae* formulated in oil achieved a 100% cumulative mortality in 6 d compared to the 8 d required for the Tween 80 formulation. This showed an increase in the virulence of the fungus by achieving a faster killing rate due to its formulation in oil. Santi et al. [17] reported formulations containing *M. anisopliae* conidia in 5% and 10% soybean oil as the most effective virulent against the cotton seed bug *Dysdercus peruvianus*. Peng and Xia [39] reported an increase in the virulence of an oil-based formulation of *M. anisopliae var. acridium* against grasshoppers and locusts by reducing the dependence on high humidity for germination, maintaining the thermotolerance and UV tolerance of the fungal conidia.

Although conidial viability was higher for *A. flavus* isolate than *M. anisopliae*, we found no significant difference in the mortality rates of the bugs between the two isolates (*p* > 0.05). However, in a previous study employing dipping bioassay involving the same isolates, a significant difference (*p* < 0.05) was observed in bug mortality [40]. The formulation of the fungal conidia in peanut oil was observed to have enhanced the virulence of both *A. flavus* isolate and *M. anisopliae* against *E. pallens*, as evidenced by their increased killing rates. Although Aspergillus species are described as facultative generalists [41], the virulence of certain species and strains against insect hosts can be enhanced to match promising biological control agents such as *M. anisopliae* when formulated in non-evaporative diluents, such as peanut oil. 

Furthermore, the LC50 values obtained for both isolates were comparable, indicating no difference in virulence between them. In a previous study, *M. anisopliae* isolate FRM515 was considered and selected as the most virulent against the brown-winged green bug, *Plautia stali*, at an LT50 value of 3.9 days. Ihara et al. [42] reported a similar LC50 value for *M. anisopliae* against adult citrus mealybug, *Planococcus citri*. There was no difference in LT50 values between the two isolates. This shows how peanut oil formulation could impact the virulence of the isolates, especially *A. flavus*, which is less often reported to demonstrate virulence against arthropods. Balogun and Fagade [35] explored an LT50 of 4 d for *Aspergillus niger* and 5 d for *M. anisopliae* against *Zonocerus variegatus*. Therefore, it is noteworthy in this study that fungal conidia formulated in peanut oil showed no significant difference in their virulence against *E. pallens* compared to the formulation in Tween 80. This implies that oil can enhance the effectiveness of these entomopathogens in terms of killing insects. 

However, the major impediment to fungal penetration is the host’s cuticle, which must be broken down to ease penetration. Fungal pathogens secrete a certain class of cuticle-degrading enzymes (protease, chitinase, and lipase) to help dissolve the host integument, thereby overcoming the barrier [43]. In a previous study, El-Sayed et al. [44] established that successful penetration and infection of the host cuticle depends on the combined outcome of enzymatic degradation and mechanical pressure exerted by the infecting fungus. For example, the lipolytic activity of CDEs has been proposed as a ‘virulence index,’ which could be harnessed for selection of effective strains from the population [45]. Recent understanding of the entomo-pathogenesis of fungi reveals that the CDE system of entomopathogenic fungi is distinctive and of considerable importance as a potential standard for improving mycoinsecticides [18].

In this study, the maximum protease activity found for both *A. flavus* and *M. anisopliae* correlated positively with the mortality rates of *E. pallens*. For *A. flavus*, protease activity was highest (2.28 U/mL) on the 8th day before decreasing. A similar observation was reported by Grewal et al. [46], observed a positive correlation between enzymatic activity and the mortality rate of *Spodoptera litura* larvae exposed to different doses of *Metarhizium rileyi*. In the present study, we recorded the highest protease activity (2.43 U/mL) for *M. anisopliae* on the 6th day, which is similar to that observed by Saleem and Ibrahim, [47], who described maximum protease activity of *M. anisopliae* (2.97 U/mL), *Beauveria bassiana* (2.61 U/mL) and *Akanthomyces lecanii* (2.49 U/mL) to be highest on the 6th day after culture. For chitinase, the maximum activity for both isolates was highest on the 8th day of culture, in agreement with the findings of Grewal et al. [48], whereas lipase enzyme maximum activity was highest for *M. anisopliae* (3.46 U/mL) on the 6th day of culture, and *A. flavus* exhibited maximum activity (3.22 U/mL) on the 8th day. A similar trend was observed by Grewal et al. [46] but contrasted by Supakdamrongkul et al. [48], who recorded the highest lipase activity of 20.8 U/mL by the 8th day from *Nomuraea rileyi*. It can be inferred from this study that the variations in maximum enzyme activities could be attributed to isolate and species types. Because arthropod integument mainly contains proteins, lipids, and chitin, the enzyme activity results obtained in this study support the ecological and physiological importance of CDEs, as previously reported [46]. Studies on the mortality of *E. pallens* and the ability of fungal isolates to produce CDE activity showed positive correlations when analyzed. This is in agreement with previous studies that reported positive correlations between mortality and CDE activity of entomopathogenic fungi [23,46,49], indicating that specific enzymes in the enzyme system may serve as key determinants of virulence.

## 4. Materials and Methods

### 4.1. E. pallens Collection and Maintenance

Adult *E. pallens* were hand-picked from under heaps of groundnut haulms in the field. The bugs were kept in a plastic container and sent to the lab. The bugs were maintained in the lab according to the method described in [40]. Adult bugs were collected for the experiment during peanut harvest between August and October 2018. *E. pallens* were identified using the molecular method [50].

### 4.2. Fungal Isolates

*Aspergillus flavus* isolate (accession number MF319893.1) (registered as ITS sequence of *A. flavus* in the NCBI databank) originally isolated from the cadavers of *E. pallens* was obtained from the Biotechnology and Applied Microbiology Laboratory of the University of Malaya, Kuala Lumpur, Malaysia. *Metarhizium anisopliae* previously reported by Balogun and Fagade [35] (originally from cadavers of Zonocerus variegatus) was collected from the Environmental Microbiology and Biotechnology Laboratory, Department of Microbiology, University of Ibadan (Ibadan, Nigeria). The purity of both fungi was checked before culturing. Pure cultures were maintained on PDA.

### 4.3. Fungal Conidia Preparation

Fungal conidia were harvested from 2-week-old culture according to the modified method of [51] by gently scrapping the culture plate with a sterile spatula to avoid dispersing the conidia into the agar. Conidia were harvested into separate sterile 50 mL centrifuge tubes, each containing 20 mL (10 mL of 5% peanut oil and 10 mL of 0.05% (*v*/*v*) Tween 80 (Sigma Chemical Co., St. Louis, MO, USA)) solution. The conidia were vigorously vortexed for 1 min to homogenize before sieving. The conidial homogenate suspensions were sieved through four layers of sterile cheesecloth into sterile centrifuge tubes and kept for further use. Conidial concentrations were estimated using a Neubauer hemocytometer under a Leica DM500 bright-field microscope (Leica Microsystems, Wetzlar, Germany) [52]. Five concentrations from 104 to 108 conidia/mL were prepared in tenfold dilutions for the bioassay. Conidial viability was determined before each bioassay by plating 100 µL aliquot of diluted conidia on three PDA plates. Three plates were prepared for each isolate with three replications and incubated at 25 °C for 24 h under 14:10 light/dark regimes. Germination was observed to be ≥90 under the microscope at 40× by counting 300 conidia (100 conidia per field). Viability was indicated for conidia that produced germ tubes half their size.

### 4.4. Dose–Response Bioassay

The dose–response bioassay was conducted using fungal pathogens on sterile filter papers according to the method described in [25] with minor modifications. For each conidial preparation, 1 mL of the suspension was pipetted onto a moist sterile filter paper (65 mm, Whatman No. 1) inside a plastic container (65 × 45 mm). Sterile cotton dipped in sterile demineralized water was added to the container to afford ≥80% relative humidity (RH) at room temperature. The bioassay tested 10 adult bugs against individual conidial concentrations inside a sterile container, with five replications for each concentration. The bugs were introduced into plastic containers containing sterile filter paper treated with 1 mL peanut oil, and Tween 80 served control. Mortality was scored at an interval of 24 h for 10 d in replicate using independent batches of bugs and conidial inoculum. Furthermore, to confirm whether the bugs’ mortality was caused by the fungal pathogen used in the bioassay, the cadavers were sterilized on the surface for 5 min using 1% (*v*/*v*) NaClO solution. The sterilized cadavers were washed 3 times in sterilized demineralized water and subjected to 5–7 d incubation at 25 ± 2 °C on sterilized wet filter paper in Petri plates to stimulate fungal growth and examined under a bright-field microscope (Leica DM500, Leica Microsystems, Wetzlar, Germany) at 400×.

### 4.5. Production of Cuticle-Degrading Enzymes

Synthesis and determination of the activity of extracellular cuticle-degrading enzymes: protease, lipase, and chitinase were studied from the isolates of *A. flavus* and *M. anisopliae* based on the method described in [53] with minor modifications. A 200 µL conidial suspension of each fungal isolate (containing 1 × 10^8^ conidia/mL) was inoculated in 100 mL of Sabouraud dextrose broth (into a 100 mL flask). Inoculated flasks were incubated on a rotary shaker at 28 °C and 150 rpm for 10 d. Enzyme assays were performed on alternate days for 10 d. The cultures were filtered using cheesecloth to remove the mycelia before centrifugation at 13,000 rpm for 10 min at 4 °C. The supernatant, the crude enzyme source, was used for the enzyme activity assay.

### 4.6. Enzyme Assays

According to the method, the protease activity was determined by employing casein as a substrate [48]. The reaction mixture (2 mL) comprised an aliquot of diluted enzyme solution, 10 mg casein, and Na_2_CO_3_ buffer (200 µM) at pH 9.7. Enzyme activity was assayed at 37 °C for 20 min and stopped by adding 3 mL trichloroacetic acid (TCA) (2.6 mL of 5% TCA and 0.4 mL of 3.3 N HCl). The absorbance of the reaction mixture was measured at 280 nm. One enzyme unit was defined as the amount of enzyme that catalyzes the release of 1 µmol of tyrosine per min under the assay conditions.

Chitinase activity was determined using a colloidal chitin substrate based on the method adopted in [54], with some modifications. Analytical-grade chitin (20 g) was suspended in 200 mL concentrated HCl and stirred for 3 min at 40 °C. Distilled water (2 L) adjusted to 5 °C was gradually added to the chitin suspension for precipitation as a colloidal suspension. The precipitated colloidal material was obtained using coarse filter paper and washed in distilled water to attain a pH of 4.0. The chitinase activity was determined using the technique described in [46]. The assay reacting mixture containing 250 µL of 0.5% (*w*/*v*) colloidal chitin, 250 µL of 0.2 acetate buffer (pH 4), and 500 µL enzyme suspension was incubated at 37 °C for 2 h. After centrifuging the resulting mixture, 0.8 M boric acid (100 µL) was mixed with the supernatant (500 µL), and the solution was adjusted to a pH of 10.2 using 1 M KOH. The solution was subjected to heat for 3 min. The resulting mixture was cooled down, and 3 mL of p-dimethyl amino benzaldehyde (DMAB) solution (1 g of DMAB dissolved in 100 mL glacial CH_3_COOH comprising 1% *v*/*v* HCl) was added before incubating at 37 °C for 20 min. Absorbance was measured at 585 nm against a water blank. A unit of enzyme activity was expressed as 1 µmol/min of N-acetylglucosamine. 

Lipase activity was determined by employing gum acacia and olive oil as substrates based on the method adopted in [46]. Substrate preparation involved mixing olive oil (50 mL) and gum acacia (50 mL, 10% *w*/*v* at 1:1 ratio). The lipase enzyme reaction mixture comprised a 5 mL substrate, 2 mL phosphate buffer (50 mM, pH 6.8), and 1 mL enzyme suspension. The reaction mixture was subjected to incubation for 1 h at 37 °C. This was followed by intermittent shaking and the inclusion of 4 mL ethanol/acetone (1:1 *v*/*v*), comprising phenolphthalein (0.09%), as an indicator to end the reaction. Lipase activity was measured by titrating the final mixture against NaOH (50 mM) solution (to estimate the free fatty acids discharged throughout the reaction). A unit of lipase enzyme activity was defined as 1 µmol fatty acids liberated per mL per min. All enzyme assays were performed in triplicate for all isolates.

### 4.7. Statistical Analyses

The experiments were performed in 5 replicates for each isolate, and the entire experiment was repeated 3 times. The mortality data were adjusted using Abbott’s formula [55]; the normality of the data was checked with a skewness–kurtosis test and analyzed using IBM SPSS Statistics 21.0 software (Long Beach, CA, USA). Analysis of variance (ANOVA) was used to make multiple comparisons. An F test was employed to measure the significant differences between treatments, and the means were compared using the least significant difference (LSD). The median lethal concentrations (LC50 and LC90) needed to kill 50% and 90% of the bugs and their respective confidence intervals (C. I.), as well as the median lethal time (LT50 and LT90), were determined with the aid of probit analysis [56]. The correlation between mortality and enzyme activity was analyzed with statistical significance set at the level of 0.05 for all tests.

## 5. Conclusions

The fungal conidial formulation demonstrated outstanding potential for controlling *E. pallens* under laboratory conditions. The oil-based conidial formulation showed enhanced virulence of both fungal isolates, implying that either of the fungi can be used as a biocontrol agent for bugs. The mortality of the bugs was observed to increase with an increase in conidial concentrations. The effectiveness of the formulated conidia at infecting and killing bugs when applied on filter papers makes these fungi potent for field applications, with a considerable possibility of success. Furthermore, the cuticle-degrading enzyme activity showed positive correlations with the mortality rates of *E. pallens* in interaction with varying concentrations of the fungal conidia. Exploiting the potential of the two fungi via inundative field applications and optimization of their enzyme production capacity will undoubtedly improve their commercial acceptance and use as biological control agents.

## Figures and Tables

**Table 1:** Mean mortality of *E. pallens* evaluated after 10 d of exposure to different formulations of entomopathogenic fungi conidia applied on sterile filter paper in the bioassay.

Oil-Formulated Conidia			Tween 80 Formulation	
Conidia/mL	Mean ± S.E.			
	*A. flavus*	*M. ansopliae*	*A. flavus*	*M. ansopliae*
10^8^	54.56 ^a^ ± 10.92	71.45 ^a^ ± 10.88	52.64 ^a^ ± 11.70	58.91 ^a^ ± 12.84
10^7^	45.91 ^a^ ±10.19	59.73 ^ab^± 10.38	39.27 ^ab^ ± 10.23	53.18 ^a^ ± 12.03
10^6^	43.46 ^a^ ± 7.06	49.64 ^bc^ ± 8.92	30.56 ^bc^ ± 9.77	48.00 ^ab^ ± 10.35
10^5^	36.27 ^ab^ ± 7.06	39.18 ^bcd^ ± 7.81	24.56 ^bc^ ± 8.36	35.82 ^bc^ ± 9.28
10^4^	31.09 ^bc^ ± 5.76	29.27 ^cd^ ± 7.30	20.00 ^bc^ ± 6.95	31.46 ^cb^ ± 8.80
Control	1.27 ^d^ ± 0.62	1.09 ^e^ ± 0.49	1.09 ^d^ ± 0.63	2.18 ^d^ ± 0.95
Skewness and kurtosis: ± 0.141 & ± 0.808				

Mean values in the same column followed by the same letters are not significantly different based on probit analysis and confidence intervals.

**Table 2:** Lethal concentration (LC_50_ and LC_90_) values for *A. flavus* isolate and *M. anisopliae* conidia formulated in oil and Tween 80 applied against *E. pallens*.

Fungal Conidia Formulated in Oil			Fungal Conidia Formulated in Tween 80	
Isolates				
	*A. flavus*	*M. anisopliae*	A. *flavus*	M. *anisopliae*
LC_50_ (conidia/mL)	1.95 × 10^6^	3.92 × 10^6^	9.36 × 10^7^	6.85 × 10^6^
95% fiducial limits (lower–upper)	2.17 × 10^4^–1.12 × 10^7^	1.86×10^4^–7.23 × 10^6^	1.37 × 10^7^–3.22 × 10^8^	1.33 × 10^6^–1.55 × 10^6^
LC_90_ (conidia/mL)	3.66 × 10^9^	5.37 × 10^8^	6.50 × 10^9^	2.57 × 10^8^
95% fiducial limits (lower–upper)	8.56 × 10^8^–11.16 × 10^9^	1.78 × 10^8^–1.45 × 10^9^	4.16 × 10^9^–1.35 × 10^12^	1.15 × 10^8^–1.24 × 10^9^
Toxicity regression equation	Y = 4.13 + 0.71 × X	Y = 4.82 + 0.82 × X	Y = 4.72 + 1.02 × X	Y = 5.21 + 0.99 × X
Slope ± standard error	0.39 ± 0.09	0.60 ± 0.16	0.48 ± 0.19	0.43 ± 0.09

X: enzyme activity.

**Table 3:** Lethal time of the virulence of fungal pathogens against *E. pallens*.

Fungal Conidia Formulated in Oil			Fungal Conidia Formulated in Tween 80	
Isolates				
	*A. flavus*	*M. anisopliae*	*A. flavus*	*M. anisopliae*
LT_50_ (days)/95% fiducial limits (lower–upper)	3.3 (1.3–4.1)	3.6 (1.3–4.2)	5.0 (4.1–5.5)	3.8 (3.1–4.2)
LT_90_ (days)/95% fiducial limits (lower–upper)	6.6 (5.9–8.1)	5.4 (5.3–6.3)	8.8 (7.6–12.1)	5.7 (5.1–6.1)
Toxicity regression equation	Y = 0.25 + 1.45 × X	Y = 0.63 + 1.66 × X	Y = 1.45 + 0.11 × X	Y = 2.97 + 1.62 × X
Slope ± standard error	0.34 ± 0.11	0.56 ± 0.16	0.44 ± 0.19	0.49 ± 0.09

Skewness and kurtosis: ± 0.143 ± 0.81.

**Table 4:** Cuticle-degrading enzyme activity of *Aspergillus flavus* isolates and *Metarhizium anisopliae*.

		Enzyme Activity on Alternate Days (U/mL) (Mean ± S.E.)					
Isolate	Enzyme	2nd Day	4th Day	6th Day	8th Day	10th Day	Mean
*A. flavus*	Protease	0.49 ± 0.14 ^a^	0.99 ± 0.20 ^ab^	1.79 ± 0.43 ^bc^	2.51 ± 0.34 ^bc^	2.02 ± 0.45 ^cd^	1.56
	Chitinase	0.63 ± 0.22 ^ab^	0.87 ± 0.03 ^ba^	0.95 ± 0.65 ^bc^	0.98 ± 0.36 ^bc^	0.37 ± 0.32 ^da^	0.76
	Lipase	1.26 ± 0.42 ^ab^	2.51 ± 0.02 ^bc^	3.22 ± 0.14 ^cb^	2.44 ±0.56 ^cb^	1.11 ± 0.65 ^dac^	2.18
*M. anisopliae*	Protease	0.68 ± 0.32 ^a^	0.94 ± 0.16 ^ba^	2.43 ± 0.28 ^cb^	2.12 ±0.12 ^cb^	1.87 ± 0.42 ^abd^	1.61
	Chitinase	0.52 ± 0.18 ^ab^	0.89 ± 0.12 ^bc^	0.92 ± 0.16 ^cb^	0.93 ±0.45 ^cb^	0.85 ± 0.54 ^ad^	0.82
	Lipase	1.09 ± 0.31 ^a^	1.59 ± 0.12 ^bc^	3.46 ± 0.23 ^cb^	2.78 ± 0.43 ^cb^	1.21 ± 0.41 ^abd^	2.02

Mean values in the same column followed by same letters are not significantly different based on probit analysis and confidence intervals.

**Table 5:** Correlation between percent mortality and enzymatic activities of the pathogens.

Entomopathogen	Protease	Chitinase	Lipase	(n)
*A. flavus*	*r* = 0.96, *p* = 0.03	*r* = 0.85, *p* = 0.19	*r* = 0.99, *p* = 0.006	27
*M. anisopliae*	*r* = 0.87, *p* = 0.16	*r* = 0.91, *p* = 0.06	*r* = 0.97, *p* = 0.04	27

## Data Availability

The data presented in this study are available in this article and Graphical Abstract.
